# Interaction between retinol intake and *ISX* rs5755368 polymorphism in colorectal cancer risk: a case–control study in a Korean population

**DOI:** 10.1038/s41598-023-36973-w

**Published:** 2023-06-22

**Authors:** Anh Quynh Bui, Madhawa Gunathilake, Jeonghee Lee, Jae Hwan Oh, Hee Jin Chang, Dae Kyung Sohn, Aesun Shin, Jeongseon Kim

**Affiliations:** 1 Department of Cancer Control and Population Health, Graduate School of Cancer Science and Policy, National Cancer Center, Goyang-si, Gyeonggi-do South Korea; 2 Department of Cancer Biomedical Science, Graduate School of Cancer Science and Policy, National Cancer Center, 323 Ilsan-ro, Ilsandong-gu, Goyang-si, 10408 Gyeonggi-do South Korea; 3 Center for Colorectal Cancer, National Cancer Center Hospital, National Cancer Center, Goyang-si, Gyeonggi-do South Korea; 4 Department of Preventive Medicine, Seoul National University College of Medicine, Jongno-gu, Seoul, South Korea

**Keywords:** Cancer epidemiology, Genetic association study

## Abstract

This study aimed to examine whether the *ISX* rs5755368 genotypes are associated with the effect of dietary retinol consumption on CRC risk. We recruited 923 CRC patients and 1846 controls to identify the association between dietary retinol and CRC risk. Dietary retinol intake was assessed using a semiquantitative food frequency questionnaire. Genotype data were available for 1419 patients (600 cases and 819 controls) of the total study population. Genotyping was performed using an Illumina MEGA Expanded Array. Odds ratios (ORs) and 95% confidence intervals (95% CIs) were calculated using unconditional logistic regression models. Retinol intake was inversely associated with CRC (OR = 0.49; 95% CI = 0.37–0.63). Participants with AA genotype showed lower CRC risk than subjects carrying the G allele (AG + GG) (OR = 0.76; 95% CI = 0.58–0.99). A 68% reduced risk of CRC was related to subjects who had the highest retinol intake and carrying AA genotype compared to the risk of participants consumed the lowest retinol intake and carrying the G allele (OR = 0.32; 95% CI = 0.20–0.53; *P* interaction = 0.026). Retinol intake could be a protective factor for CRC risk while this association could be strengthened among individuals carrying the homozygous AA genotype.

## Introduction

According to the GLOBOCAN cancer statistics, in 2020, colorectal cancer (CRC) was reported as the third most common cancer worldwide and the second most common cancer in terms of cancer mortality, with over 1.9 million new cases and 935,000 deaths^1^. In South Korea, the latest cancer data revealed that the fourth-highest cancer incidence rate was identified in CRC among both sexes, which was estimated to be 28.4 cases per 100,000 persons per year^2^. In addition, the CRC mortality rate ranks third after lung and liver cancer deaths in Korea^2^. The World Cancer Research Fund/American Institute for Cancer Research suggested an association between several dietary factors and the risk of CRC, namely, whole grains, fiber, dairy products and calcium, red meat and processed meat, vitamin C, vitamin D, and multivitamin supplements^3^. However, the evidence for the protective role of vitamin A against CRC development is controversial^4–6^.

Retinol is the most biologically active form of vitamin A^7^. The main sources of dietary retinol are liver, egg yolk and milk fat^8^. Retinol influences cell differentiation, proliferation, and apoptosis and plays an important physiologic role in a wide range of biological processes^9^. In several studies, retinol was reported as a potential factor associated with cancer prevention, including lung cancer^7,10^, cervical cancer^11^, esophageal cancer^12^, and gastric cancer^13,14^. However, the protective effect of retinol in CRC is still inconsistent. While an inverse association between retinol and CRC risk was uncovered in some previous studies^15–17^, others have revealed that retinol consumption is not significantly associated with CRC prevention^18,19^.

Homeobox genes are an evolutionarily conserved class of transcription factors that are key regulators during developmental processes such as regional specification, patterning, and differentiation^20^. Deregulation of homeobox genes enhances cell survival and proliferation and inhibits cell differentiation^21^. The intestine-specific homeobox (*ISX*) gene is a member of the homeobox superfamily^22^. Pathologic studies have revealed that *ISX* exhibits a tumour-specific expression pattern and highly correlates with patient survival as well as tumour size, number, and stage^23^. While it is a well-known proto-oncogene involved in the development of hepatocellular carcinoma (HCC)^23–25^, gastric cancer^26^, pancreatic cancer^27^, and lung cancer^28^, the function of *ISX* in CRC is unclear. In addition, in vivo studies have revealed that *ISX* expression is regulated by retinoic acid (RA)^29^, a metabolite of retinol^30^. Thus, we considered whether retinol intake could affect the function of *ISX* genetic variations and contribute to CRC development. However, there is no evidence for an interaction between retinol and genetic variants in the development of CRC. A single nucleotide polymorphism (SNP) of the *ISX* gene, rs5755368, is associated with β-carotene absorption in the intestine^31^, suggesting that it might affect retinoid production and storage in the human body. Therefore, we conducted a case–control study to examine the association between dietary retinol intake and the risk of CRC. Furthermore, we investigated whether the association between retinol intake and CRC risk could be modified by the *ISX* rs5755368 polymorphism.

## Results

### Demographic characteristics of the study participants

Table 1 shows the general characteristics of the subjects in this study. There were significant differences between the CRC patients and the controls with respect to body mass index (BMI) (kg/m^2^), the rate of family history of CRC (FHCC), and alcohol consumption in the total population (p < 0.001) and among men (p < 0.001). However, a higher proportion of current smokers in the CRC group than in the controls was significant only among women (p < 0.001). In addition, the cases were likely to have a lower physical activity frequency (p < 0.001), education level (p < 0.001), monthly income (p < 0.001), and professional occupation (p < 0.001). Regarding dietary intake in the study population, the total energy intake in the CRC group was significantly higher than that in the control group(p < 0.001). In contrast, the CRC patients tended to have a lower total retinol intake than the controls, which was reported to be 60.1 ± 39.5 µg/day and 78.7 ± 55.4 µg/day (p < 0.001), respectively. We also report that some retinol-contributing foods, including eggs (p < 0.001), cow’s milk (p < 0.001), eel (p < 0.001), beef (p < 0.001), pork (p < 0.005), cheese (p < 0.001), and yogurt (p < 0.05), were highly consumed by the CRC-free participants compared to the cases among both sexes and even after stratification by sex (Supplementary Table 1).

**Table 1:** Demographic characteristics of the study participants.

Characteristics	Total (n = 2769)			Men (n = 1875)			Women (n = 894)		
	Control (n = 1846)	Case (n = 923)	p-value^a^	Control (n = 1250)	Case (n = 625)	p-value^a^	Control (n = 596)	Case (n = 298)	p-value^a^
Age (years)									
Mean ± SD	56.1 ± 9.1	56.6 ± 9.7	0.200	56.6 ± 8.7	57.2 ± 9.4	0.205	54.9 ± 9.8	55.3 ± 10.2	0.643
Missing	0 (0)	0 (0)		0 (0)	0 (0)		0 (0)	0 (0)	
BMI (kg/m^2^, n, %)									
Mean ± SD	24.1 ± 2.7	23.6 ± 3.4	** < 0.001**	24.4 ± 2.6	23.6 ± 3.1	** < 0.001**	23.3 ± 2.8	23.7 ± 4.0	0.142
< 25	1215 (65.8)	640 (69.3)	**0.016**	766 (61.3)	433 (69.3)	** < 0.001**	449 (75.3)	207 (69.5)	0.064
≥ 25	620 (33.6)	283 (30.7)		476 (38.1)	192 (30.7)		144 (24.2)	91 (30.5)	
Missing	11 (0.6)	0 (0)		8 (0.4)	0 (0)		3 (0.5)	0 (0)	
Educational status (n, %)									
Middle school or less	282 (15.3)	321 (34.8)	** < 0.001**	175 (14.0)	183 (29.3)	** < 0.001**	107 (17.9)	138 (46.3)	** < 0.001**
High school	587 (31.8)	369 (40.0)		329 (26.3)	266 (42.6)		258 (43.3)	103 (34.6)	
College or more	934 (50.6)	233 (25.2)		712 (57.0)	176 (28.2)		222 (37.3)	57 (19.1)	
Missing	43 (2.3)	311 (33.7)		34 (2.7)	0 (0)		9 (1.5)	0 (0)	
Family history of colorectal cancer (n, %)									
No	1743 (94.4)	837 (90.7)	** < 0.001**	1188 (95.0)	560 (89.6)	** < 0.001**	555 (93.1)	277 (92.9)	0.926
Yes	99 (5.4)	86 (9.3)		58 (4.6)	65 (10.4)		41 (6.9)	21 (7.1)	
Missing	4 (0.2)	0 (0)		4 (0.3)	0 (0)		0 (0)	0 (0)	
Regular exercise (n, %)									
No	753 (40.8)	612 (66.3)	** < 0.001**	489 (39.1)	387 (61.9)	** < 0.001**	264 (44.3)	225 (75.5)	** < 0.001**
Yes	1047 (56.7)	311 (33.7)		716 (57.3)	238 (38.1)		331 (55.5)	73 (24.5)	
Missing	46 (2.5)	0		45 (3.6)	0		1 (0.2)	0	
Smoking status (n, %)									
Non-smoker	818 (44.3)	409 (44.3)	0.156	247 (19.8)	145 (23.2)	0.082	571 (95.8)	264 (88.6)	** < 0.001**
Ex-smoker	687 (37.2)	318 (34.5)		671 (53.7)	303 (48.5)		16 (2.7)	15 (5.0)	
Current smoker	341 (18.5)	196 (21.2)		332 (26.6)	177 (28.3)		9 (1.5)	19 (6.4)	
Missing	0 (0)			0 (0)	0 (0)		0 (0)	0 (0)	
Alcohol consumption (n, %)									
Non-drinker	560 (30.3)	279 (30.2)	** < 0.001**	200 (16.0)	107 (17.1)	**0.001**	360 (60.4)	172 (57.7)	0.191
Ex-drinker	169 (9.2)	129 (14.0)		136 (10.9)	103 (16.5)		33 (5.5)	26 (8.7)	
Current drinker	1117 (60.5)	515 (55.8)		914 (73.1)	415 (66.4)		203 (34.1)	100 (33.6)	
Missing	0	0		0	0		0	0	
Marital status (n, %)									
Married	1654 (89.6)	773 (83.8)	** < 0.001**	1161 (92.9)	557 (89.1)	** < 0.001**	493 (82.7)	216 (72.5)	**0.001**
Others	171 (9.3)	146 (15.8)		73 (5.8)	66 (10.6)		98 (16.4)	80 (26.9)	
Missing	21 (1.1)	4 (0.4)		16 (1.3)	2 (0.3)		5 (0.8)	2 (0.7)	
Monthly average gross income (10,000 won/moth) (n, %)									
< 200	388 (21.0)	321 (34.8)	** < 0.001**	254 (20.3)	222 (35.5)	** < 0.001**	134 (22.5)	99 (33.2)	** < 0.001**
200–400	754 (40.9)	387 (41.9)		536 (42.9)	253 (40.5)		218 (36.6)	134 (45.0)	
> 400	545 (29.5)	215 (23.3)		362 (29.0)	150 (24.0)		183 (30.7)	65 (21.8)	
Missing	159 (8.6)	0 (0)		98 (7.8)	0 (0)		61 (10.2)	0 (0)	
Occupation (n, %)									
Professional administrative, office jobs	481 (26.1)	189 (20.5)	** < 0.001**	389 (31.1)	160 (25.6)	** < 0.001**	92 (15.4)	29 (9.7)	** < 0.001**
Sale and service positions	403 (21.8)	38 (4.1)		308 (24.6)	28 (4.5)		95 (15.9)	10 (3.4)	
Agriculture, manufacturing, mining, army service	241 (13.1)	141 (15.3)		221 (17.7)	121 (19.4)		20 (3.4)	20 (6.7)	
Housekeeping, unemployment, and others	698 (37.8)	555 (60.1)		314 (25.1)	316 (50.6)		384 (64.4)	239 (80.2)	
Missing	23 (1.3)	0 (0)		18 (1.4)	0 (0)		5 (0.8)	0 (0)	
Total energy intake (kcal/day)	1689.6 ± 560.4	2026.3 ± 534.0	** < 0.001**	1730.4 ± 547.2	2127.4 ± 509.1	** < 0.001**	1604.0 ± 578.4	1814.4 ± 523.5	** < 0.001**
Retinol intake^b^ (µg/day)	78.7 ± 55.4	60.1 ± 39.5	** < 0.001**	73.7 ± 49.2	55.9 ± 35.4	** < 0.001**	89.2 ± 65.4	69.0 ± 45.8	** < 0.001**

Significance at p-value < 0.05.

Significant values are in bold.

^a^p-values were calculated using the Chi-square test for categorical variables and the t-test for continuous variables.

^b^Retinol intake was adjusted for total energy intake using the residual method.

### Association between retinol intake and CRC risk

In Table 2, the association of retinol intake with risk of CRC is expressed as odds ratios (ORs) and 95% confidence intervals (CIs). Retinol intake on CRC risk was divided into tertiles to compare CRC risk across each tertile. Higher retinol consumption was inversely associated with a reduced risk of CRC (OR = 0.39; 95% CI = 0.32–0.48; *P* for trend < 0.001). This association remained in the multivariate model adjusted for age, sex, BMI, education level, occupation, monthly income, smoking status, alcohol consumption, physical activity, FHCC, and total energy intake (OR = 0.49; 95% CI = 0.37–0.63; *P* for trend < 0.001). When stratifying by sex, a statistical relationship between retinol intake and the risk of CRC was observed among men (OR = 0.43; 95% CI = 0.31–0.60; *P* for trend < 0.001). However, there was no significant difference in the CRC risk of the female patients compared to the female controls in terms of retinol intake after adjusting for confounders.

**Table 2:** Association between retinol intake and CRC risk.

Retinol intake (µg/day)	Controls	Cases	Model 1 [OR (95% CI)]	Model 2 [OR (95% CI)]
Total (n = 2769)				
T1 (< 48.75)	616 (33.4)	443 (48.0)	1.00	1.00
T2 (48.75–88.17)	615 (33.3)	306 (33.2)	0.69 (0.58–0.83)	0.68 (0.54–0.86)
T3 (> 88.17)	615 (33.3)	174 (18.9)	0.39 (0.32–0.48)	0.49 (0.37–0.63)
* p* for trend			< 0.001	< 0.001
Men (n = 1875)				
T1 (< 47.20)	417 (33.4)	317 (50.7)	1.00	1.00
T2 (47.20–83.41)	416 (33.3)	191 (30.6)	0.60 (0.48–0.76)	0.58 (0.43–0.77)
T3 (> 83.41)	417 (33.4)	117 (18.7)	0.37 (0.29–0.48)	0.43 (0.31–0.60)
* p* for trend			< 0.001	< 0.001
Women (n = 894)				
T1 (< 51.54)	198 (33.2)	124 (41.6)	1.00	1.00
T2 (51.54–98.79)	199 (33.4)	116 (38.9)	0.93 (0.68–1.28)	1.14 (0.75–1.72)
T3 (> 98.79)	199 (33.4)	58 (19.5)	0.47 (0.32–0.67)	0.73 (0.46–1.15)
* p* for trend			< 0.001	< 0.001

Model 1: crude model. Model 2: multivariate model, adjusted for age, sex, body mass index (BMI), family history of colorectal cancer, smoking status, alcohol consumption, regular exercise, education, occupation, monthly income, married status, and total energy intake.

*T* tertile (µg/day).

We also examined whether the difference in the FHCC status among the study groups affected the association between dietary retinol consumption and CRC occurrence (Supplementary Table 2). In the participants without a FHCC, a decreased risk of CRC was reported in those consuming a higher level of retinol even after adjusting for potential confounders (OR = 0.49; 95% CI: 0.38–0.65; *P* for trend < 0.001). Similarly, this inverse association was also investigated among the FHCC group (OR = 0.28; 95% CI = 0.09–0.84), but the trend was not significant (p > 0.05).

### Association of *ISX* rs5755368 polymorphism with CRC risk

Table 3 shows the association between the *ISX* rs5755368 A > G polymorphism and the risk of CRC. The *ISX* rs5755368 minor allele frequency is G = 0.20 among the Korean population^32^. Polymorphism in the controls was in Hardy–Weinberg equilibrium (p = 0.175). Participants carrying the homozygous AA genotype had a 46% lower risk of CRC compared to those who carried the G allele (OR = 0.54; 95% CI = 0.30–0.97; p = 0.038), suggesting that the AA genotype of the *ISX* rs5755368 polymorphism may be a protective factor against CRC occurrence. This evidence was more explicit when we further analyzed the dominant model, as the risk of CRC in the participants carrying the homozygous AA genotype was significantly lower than that in the subjects carrying the G allele (AG + GG), even after adjusting for multiple variables (OR = 0.76; 95% CI = 0.58–0.99; p = 0.045).

**Table 3:** Association between *ISX* rs5755368 polymorphism and colorectal cancer risk.

*ISX* rs5755368	Controls	Cases	Model 1			Model 2		
			OR	95% CI	p-value^a^	OR	95% CI	p-value^a^
GG	22 (2.7)	27 (4.5)	1.00			1.00		
AG	258 (31.5)	215 (35.8)	0.68	0.38–1.23	0.199	0.70	0.34–1.47	0.352
AA	539 (65.8)	358 (59.7)	0.54	0.30–0.97	0.038	0.55	0.27–1.14	0.108
Dominant								
AG + GG	280 (34.2)	242 (40.3)	1.00			1.00		
AA	539 (65.8)	358 (59.7)	0.77	0.62–0.96	0.018	0.76	0.58–0.99	0.045
Recessive								
GG	22 (2.7)	27 (4.5)	1.00			1.00		
AA + AG	797 (97.3)	573 (95.5)	0.59	0.33–1.04	0.067	0.60	0.29–1.24	0.168

rs5755368 polymorphism A > G was available among 600 cases and 819 controls. Model 1: crude model. Model 2: multivariate model, adjusted for age, sex, body mass index (BMI), family history of colorectal cancer, smoking status, alcohol consumption, regular exercise, education, occupation, monthly income, married status, and total energy intake.

^a^p-values were calculated using the Chi-square test.

### Interaction between retinol intake and *ISX* rs5755368 polymorphism in CRC risk

As the retinol intake and the *ISX* rs5755368 polymorphism were significantly associated with the risk of CRC, we considered the interaction between retinol intake and the rs5755368 genetic polymorphism in CRC occurrence. Table 4 shows that the homozygous AA participants who consumed a higher retinol intake were likely to have a considerably lower risk of CRC regardless of the abovementioned confounding factors in the dominant model (OR = 0.32; 95% CI = 0.20–0.53; *P* for interaction = 0.026). Males carrying homozygous major allele who had a higher intake of dietary retinol showed a significantly reduced risk of CRC compared to those carrying the G allele (AG or GG genotypes) and consuming a lower retinol intake (OR = 0.28; 95% CI = 0.15–0.52). However, a significant interaction was not observed (*P* for interaction = 0.086). There was no statistical interaction of the G allele and dietary retinol in the reduced risk of CRC.

**Table 4:** Interaction between retinol intake and *ISX* rs5755368 polymorphism (dominant model) in colorectal cancer risk**.**

*ISX* rs5755368	AG/GG			AA			*P* interaction
Total (n = 1419)	T1 (< 48.75)	T2 (48.75–88.17)	T3 (> 88.17)	T1 (< 48.75)	T2 (48.75–88.17)	T3 (> 88.17)	
No. of controls/cases	97/114	94/75	89/53	167/176	193/127	179/55	
Model 1 [OR (95% CI)]	1.00	0.68 (0.45–1.02)	0.51 (0.33–0.78)	0.90 (0.64–1.27)	0.56 (0.39–0.80)	0.26 (0.17–0.39)	0.076
Model 2 [OR (95% CI)]	1.00	0.77 (0.47–1.26)	0.76 (0.45–1.30)	0.97 (0.64–1.48)	0.66 (0.43–1.01)	0.32 (0.20–0.53)	0.026

Men (n = 944)	T1 (< 47.20)	T2 (47.20–83.41)	T3 (> 83.41)	T1 (< 47.20)	T2 (47.20–83.41)	T3 (> 83.41)	
No. of controls/cases	67/85	61/40	60/43	110/116	121/84	119/38	
Model 1 [OR (95% CI)]	1.00	0.52 (0.31–0.86)	0.57 (0.34–0.94)	0.83 (0.55–1.26)	0.55 (0.36–0.84)	0.25 (0.16–0.41)	0.116
Model 2 [OR (95% CI)]	1.00	0.54 (0.28–1.05)	0.69 (0.37–1.30)	0.94 (0.55–1.59)	0.67 (0.39–1.15)	0.28 (0.15–0.52)	0.086

Women (n = 475)	T1 (< 51.54)	T2 (51.54–98.79)	T3 (> 98.79)	T1 (< 51.54)	T2 (51.54–98.79)	T3 (> 98.79)	
No. of controls/cases	32/28	28/30	32/16	56/58	76/41	57/21	
Model 1 [OR (95% CI)]	1.00	1.22 (0.59–2.52)	0.57 (0.26–1.25)	1.18 (0.63–2.21)	0.62 (0.33–1.16)	0.42 (0.21–0.86)	0.264
Model 2 [OR (95% CI)]	1.00	1.43 (0.60–3.44)	1.47 (0.55–3.93)	1.02 (0.47–2.23)	0.79 (0.36–1.75)	0.69 (0.29–1.67)	0.196

Model 1: crude model. Model 2: multivariate model, adjusted for age, sex, body mass index (BMI), family history of colorectal cancer, smoking status, alcohol consumption, regular exercise, education, occupation, monthly income, married status, and total energy intake.

*T* tertile (µg/day).

## Discussion

This study indicated that retinol intake was inversely associated with the risk of CRC among both sexes. Additionally, we suggested a potential interaction of dietary retinol and the *ISX* rs5755368 homozygous AA genotype with regard to CRC occurrence.

To date, the association between retinol intake and CRC risk is controversial. Several observational studies have reported that retinol is a protective nutrient against CRC initiation^15–17,33^. However, the studies of Key et al.^18^, Kabat et al.^19^, and Shin et al.^34^ revealed that this relationship did not exist. These studies indicated that a limitation in their ability to detect any effect of retinol intake on CRC risk was the small number of cases, and random error in the dietary assessment may have existed in the study of Shin et al.^34^. In addition, after sex and FHCC status stratification, the protective role of retinol against CRC remained significant only among men and subjects without FHCC status. The unbalanced number of each comparative group could be an appropriate explanation for the null association.

Although there were inconsistent results, we could consider that there is a potential effect of retinol on CRC development. Some studies have reported the role of retinol in the prevention of CRC with plausible mechanisms. Dietary retinol can be absorbed into cells via stimulation by retinoic acid 6 or diffuse through the cell membrane. Intracellularly, retinol can be stored as retinyl esters or converted to all-trans-retinoic acid (ATRA)^35^. ATRA is critically important for gene expression because it is involved in transcription, cell signaling, and tumour suppression^36^. A recent review concluded that colorectal tumours often lose the ability to synthesize ATRA from vitamin A (retinol) and exhibit increased degradation of ATRA to 4-oxo-retinoic acid^35^. Another way that dietary retinol protects against CRC initiation is through retinoid activity. Retinoids, derivatives of vitamin A (retinol), decrease signaling via the major pathways that promote CRC progression, such as Wnt/β-catenin signaling, K-ras mutations, phosphatidylinositol-3-kinase/Akt, cyclooxygenase-2 overexpression, peroxisome proliferator-activated receptor γ activation, and loss of p53 function^35^.

The association between the *ISX* gene and cancer was reported in previous studies. Most of them suggested that *ISX* expression is a genetic factor of HCC development. Hsu et al. revealed that ablation of *ISX* in hepatoma cells suppressed cell growth, whereas overexpression promoted cell proliferation and led to enhanced tumorigenic activity in vitro and in vivo^37^. Currently, two other studies demonstrated that *ISX* is involved in a positive feedback loop, including inflammation, tryptophan catabolism, and immune suppression. Interleukin-6 induces the transcriptional activation of *ISX* to promote the production of the tryptophan catabolic enzymes tryptophan 2,3-dioxygenase and indoleamine 2,3-dioxygenase 1 in HCC. Both enzymes increase the levels of tryptophan catabolites, kynurenine, and aryl hydrocarbon receptors and activate the kynurenine/aryl hydrocarbon receptor axis. Activation of this axis promotes a positive feedback mechanism to increase *ISX*-associated proliferation, tumorigenesis, and immunotolerance^38,39^. Therefore, these studies have indicated that *ISX* was closely associated with the prognosis of patients with HCC. Patients with HCC having relatively lower *ISX* expression had a significantly longer survival time than patients with HCC having relatively higher expression after liver resection^39^. Additionally, the essential role of the *ISX* gene in hepatoma tumour formation was reported in the study of Hsu et al.^25^. Through direct regulation of cyclin D1 and E2F1 expression, *ISX* regulated the proliferation of tumour cells and their transforming activity^25^. Apart from HCC development, *ISX* expression was associated with the risk of gastric cancer, lung cancer, and the survival of pancreatic cancer patients. *ISX* expression induced by *Helicobacter pylori* infection may lead to intestinal metaplasia and hyperproliferation of gastric carcinogenesis by activating the NF-κB pathway^26^. In lung cancer, *ISX* is acetylated by the presence of p300/CBP-associated factor and then interacts with acetylated bromodomain‐containing protein 4 to regulate tumour initiation and metastasis^28^. A study by Ganaie et al.^27^ identified that high *ISX* expression correlated with poor survival of pancreatic cancer patients, particularly among women. They also suggested *ISX* as a progression biomarker and regulator of the metastasis process in pancreatic cancer disease^27^.

The *ISX* gene is a new member of the homeobox superfamily, and the evidence of its effect on cancer aetiology has been carefully evaluated. However, the association of this genetic factor with CRC development is still limited. To our knowledge, only one study by Kim et al. has identified the potential role of *ISX* expression in CRC survival^40^. They identified the *ISX* gene as a predictive marker of recurrence or metastasis in CRC patients who were treated with cetuximab regimens based on the expression of the rs361863 polymorphism^40^. The main point was that patients carrying the common allele of *ISX* rs361863 (homozygous CC genotype) seem to exhibit longer progression-free survival than that of individuals carrying heterozygous or homozygous substitution alleles (CT + TT)^40^. Although the result was not statistically significant, this SNP could be considered to improve treatment responses. Interestingly, a similar trend was shown in our study. Additionally, we found a decreased CRC risk associated with homozygous AA compared with the G allele (AG + GG) of the *ISX* rs5755368 polymorphism.

In addition to the effect of each mentioned environmental and genetic factor on CRC development, our study demonstrated a significant interaction between the rs5755368 genotypes and retinol intake in reducing the risk of CRC. Homozygous AA subjects who consumed the highest level of retinol intake were less likely to develop CRC compared to those carrying the G allele who consumed the lowest retinol intake. Additionally, we could consider this potential interaction in the human body with biological plausibility. First, *ISX* expression is regulated by vitamin A-derived retinoids^29^. RA via retinoic acid receptors (RARs) induces expression of the *ISX* gene. Lobo et al. analyzed this dependency using mouse models, and they observed that in animals subjected to vitamin A depletion through a diet lacking any source of vitamin A, *ISX* mRNA levels were decreased, thus confirming the vitamin A dependency of *ISX* mRNA expression^29^. Second, the transcription factor *ISX* suppresses the gene expression of scavenger receptor class B type 1 and β-carotene-15,15’-dioxygenase (BCO1), which encode proteins that mediate the uptake of carotenoids and their conversion into retinoids^29,41–44^. Previous studies have reported the effect of *ISX* in regulating vitamin A production by using *ISX*-deficient mouse models. Widjaja et al.^44^ generated *ISX*-deficient mice and observed that loss of this genetic factor affected retinoid metabolism in the intestine as well as systemically. *ISX* knockout mice displayed highly elevated intestinal mRNA and protein levels of BCO1 and produced significantly higher amounts of vitamin A from β-carotene supplementation than wild-type mice^43^. This evidence supported that *ISX* acts as an RA-sensitive “gatekeeper” that controls vitamin A production by repressing the gene expression of scavenger receptor class B type 1 and BCO1^29^. Vitamin A is a limited nutrient, and an uncontrolled amount of vitamin A is associated with disease; in particular, excess vitamin A can potentially promote inflammatory disorders^45^. Thus, the utilization of β-carotene, which exists in quite significant amounts in many fruits and vegetables (50–100 mg/kg), must be controlled to prevent the development of inflammatory diseases^44^. In a mouse trial, Widjaja et al. found that *ISX*-deficient mice developed pancreatic insulitis, which suggested that loss of the *ISX*-dependent control of provitamin A metabolism triggered this pathology ^44^. In summary, *ISX* expression is critical for controlling the excessive amount of dietary vitamin A.

Our study is the first investigation to report the *ISX* rs5755368 polymorphism as a genetic factor for CRC risk. Additionally, we suggested an interaction between retinol intake and the rs5755368 homozygous AA genotype in CRC risk reduction. However, there are some limitations in this study. First, the number of participants carrying homozygous GG genotype of *ISX* rs5755368 polymorphism was relatively small and may be one reason why the effect of the G allele on the inverse association between retinol intake and CRC etiology was inconclusive. In addition, as this study consisted of a hospital-based control group, it might not represent a CRC-free Korean population. Recall bias may exist when interviewing the participants using a semiquantitative food frequency questionnaire (SQFFQ). The generalizability of the current results has to be cautiously considered since no validation study has been conducted using another Korean population or a different population that represents another ethnicity. Thus, further studies are needed to confirm the proposed interaction between retinol intake and the *ISX* rs5755368 polymorphism in CRC risk.

The current study suggested that a higher retinol intake among individuals with the homozygous AA genotype of the *ISX* rs5755368 polymorphism may be involved in CRC risk reduction. This finding supports the hypothesis that genetic variation could be an important risk factor in the individual responses to a particular diet for cancer prevention.

## Methods

### Study population

**Figure 1:**
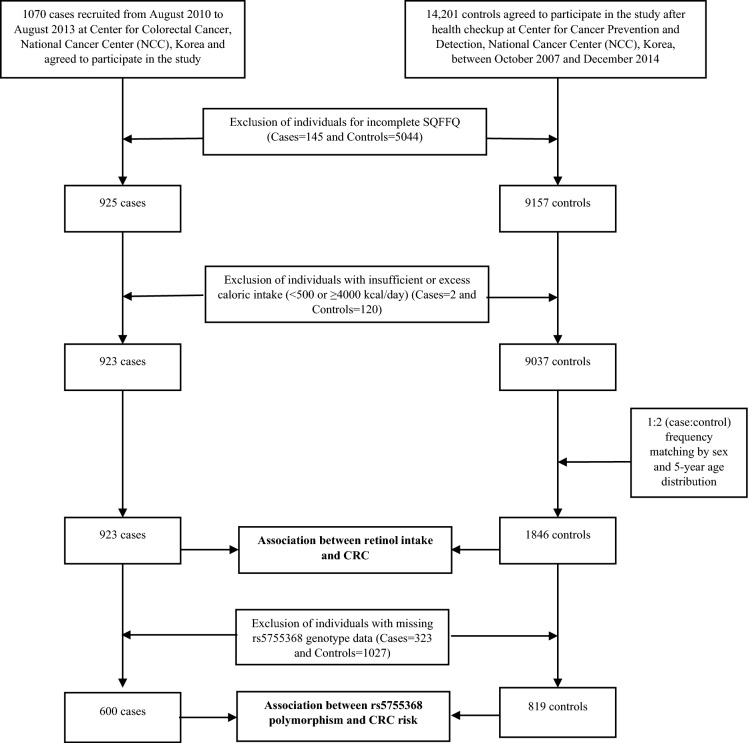
Flowchart of selecting study participants in this case–control.

This hospital-based case–control study was conducted to examine the interaction between nutritional factors and genetic variations in the risk of CRC occurrence among the Korean population, which was described elsewhere^46^. Briefly, a total of 1070 eligible CRC patients were initially recruited from August 2010 to August 2013 at the Center for Colorectal Cancer, Korean National Cancer Center (KNCC), and agreed to participate in the study. After excluding 147 subjects [who submitted an incomplete SQFFQ or whose energy intake was insufficient or excessive (< 500 or ≥ 4000 kcal/day)], the current study included 923 CRC patients. For the control group, we contacted visitors who underwent a health examination at the Center for Cancer Prevention and Detection, KNCC, between October 2007 and December 2014. Among the visitors, 14,201 subjects agreed to participate in this study. After the exclusion of 5044 subjects with an incomplete SQFFQ and 120 individuals with an implausible calorie intake, the number of potential controls was 9037 patients. Among the eligible controls, 1846 participants were selected by 1:2 frequency matching based on sex and a 5-year age distribution to examine the association between retinol intake and CRC risk. For genetic analysis, 323 cases and 1027 controls with missing *ISX* rs5755368 genotype data were excluded. Finally, we studied 600 cases and 819 controls to evaluate the effects of a genetic polymorphism (rs5755368) on CRC initiation and to identify any interaction between this SNP and retinol intake in the etiology of CRC (Fig. 1). This study conformed to the KNCC guidelines, and participants signed a written informed consent document. The Institutional Review Board of the Korean National Cancer Center approved the study (IRB Nos. NCCNCS-10–350 and NCC2015-0202).

### Data collection

The study participants were interviewed to collect their demographic and lifestyle information following a structured questionnaire. To collect dietary intake data from the study participants, a validated 106-item SQFFQ was used by a trained interviewer^47^. Total caloric and retinol intakes were calculated using the Computer Aided Nutritional Analysis Program 4.0 (CAN-PRO 4.0, The Korean Nutrition Society, Seoul, Korea).

### SNP genotyping

Genomic DNA was extracted from participants’ blood samples following the manufacturer’s instructions using the MagAttract DNA Blood M48 Kit (Qiagen, Hilden, Germany) and BioRobot M48 automatic extraction equipment (Qiagen). SNP genotyping was performed using an Illumina MEGA-Expanded Array (Illumina Inc., CA, USA) including 123K SNPs. This methodology was described elsewhere^48^.

### Statistical analyses

The general characteristics of the study population were analyzed by using the χ^2^ test for categorical variables and Student’s t-test for continuous variables. Retinol consumption was adjusted for total energy intake using the residual method. To examine its association with CRC incidence, we categorized retinol intake into tertiles based on its distribution in the control group and calculated ORs and 95% CIs using unconditional logistic regression models. We also analyzed a multivariate model adjusted for potential confounding factors, including age, sex, BMI, education level, occupation, monthly income, smoking status, alcohol consumption, physical activity, FHCC, and total energy intake. Regarding genetic analyses, rs5755368 was in Hardy–Weinberg equilibrium, and genotype was categorized into dominant (AA versus AG + GG) and recessive (AA + AG versus GG) effect models. ORs and 95% CIs were calculated to demonstrate whether there was an interaction between retinol intake and the rs5755368 polymorphism relevant to the CRC risk by using the likelihood ratio test between the models with and without the interaction term (retinol*SNP), adjusting for the abovementioned variables. Data were analyzed by using SAS version 9.4 software (SAS Institute Inc., Cary, NC, USA), and statistical significance was set at p < 0.05.

## Supplementary Information

Supplementary Tables.

## Data Availability

The variant data for this study have been deposited in the European Variation Archive (EVA) at EMBL-EBI under accession number PRJEB60396. (https://www.ebi.ac.uk/eva/?eva-study=PRJEB60396).
