# Supplementary material for: Interaction between retinol intake and ISX rs5755368 polymorphism in colorectal cancer risk: a case–control study in a Korean population
Source: Sci Rep. 2023 Jun 22;13:10187. doi: 10.1038/s41598-023-36973-w (PMC10287678; doi:10.1038/s41598-023-36973-w)
Supplement: Supplementary file 1 — Supplementary Tables. [file 41598_2023_36973_MOESM1_ESM.pdf]

Food consumption (g/d)	Cumulative (%)[a]	Total (n = 2769)			Men (n = 1875)			Women (n = 894)		
		Control (n=1846)	Case (n=923)	*p*-value[b]	Control (n=1250)	Case (n=625)	*p*-value[b]	Control (n=596)	Case (n=298)	*p*-value[b]
Hen's egg	35.78	19.5 ± 21.0	14.4 ± 14.5	**<0.001**	18.7 ± 19.9	13.5 ± 12.0	**<0.001**	21.2 ± 23.0	16.2 ± 18.4	**0.000**
Cow's milk	52.59	182.8 ± 693.0	49.0 ± 164	**<0.001**	138.8 ± 651.6	28.4 ± 69.9	**<0.001**	275.3 ± 765.0	92.2 ± 265.4	**<0.001**
Ice cream	61.21	333.5 ± 5149.4	72.7 ± 1195.1	**0.039**	141.8 ± 2444.3	64.9 ± 1364.0	0.383	735.5 ± 8333.2	88.9 ± 724.1	0.061
Eel	67.04	1.5 ± 7.2	0.4 ± 1.5	**<0.001**	1.3 ± 4.2	0.4 ± 1.3	**<0.001**	2.0 ± 11.1	0.4 ± 1.8	**0.001**
Beef meat	71.53	23.2 ± 18.1	17.3 ± 12.6	**<0.001**	23.8 ± 18.8	17.0 ± 13.0	**<0.001**	21.9 ± 16.3	18.0 ± 11.6	**<0.001**
Pork meat	74.93	40.9 ± 54.8	28.5 ± 34.4	**<0.001**	43.1 ± 55.0	29.0 ± 33.4	**<0.001**	36.4 ± 54.3	27.3 ± 36.3	**0.003**
Cheese	77.90	2.4 ± 10.2	0.5 ± 3.7	**<0.001**	1.6 ± 5.1	0.2 ± 1.6	**<0.001**	4.0 ± 16.2	1.2 ± 6.0	**<0.001**
Mackerel	80.40	8.1 ± 19.4	7.2 ± 11.2	0.097	7.0 ± 12.6	6.1 ± 8.8	0.067	10.4 ± 28.7	9.4 ± 14.7	0.461
Chicken meat	82.72	19.0 ± 98.4	9.0 ± 60.7	**0.001**	15.8 ± 83.9	6.6 ± 43.8	**0.002**	25.8 ± 123.1	13.9 ± 85.9	0.095
Yoghurt	84.79	235.7 ± 2497.9	29.6 ± 104.9	**<0.001**	211.4 ± 2834.8	15.5 ± 48.2	**0.015**	286.8 ± 1573.1	59.4 ± 167.3	**0.001**
Castella	86.78	16.3 ± 227.8	5.5 ± 54.9	0.054	8.7 ± 52.9	4.4 ± 61.5	0.135	32.2 ± 393.3	7.8 ± 37.6	0.134
Cakes	88.41	9.3 ± 87.6	3.5 ± 46.8	**0.025**	7.9 ± 74.9	3.5 ± 54.9	0.153	12.1 ± 109.6	3.6 ± 21.6	0.066
Cereals	89.88	0.5 ± 2.8	0.3 ± 1.9	**0.009**	0.4 ± 2.7	0.2 ± 1.7	**0.019**	0.7 ± 2.9	0.4 ± 2.1	0.203
Quail's egg	90.98	0.2 ± 0.3	0.1 ± 0.2	**<0.001**	0.2 ± 0.3	0.1 ± 0.1	**<0.001**	0.2 ± 0.3	0.2 ± 0.2	**<0.001**

Supplementary table 1. Comparison of the consumption of retinol contribution foods adjusted for total energy intake using residual method. [a] Food items contributing to retinol that represented up to 90% of the cumulative contribution were selected. [b] *p*-value was calculated by t-test.

**Retinol intake (μg/day)**	**Controls (n=1846)**	**Cases (n=923)**	**Model 1 [OR (95% CI)]**	**Model 2 [OR (95% CI)]**
**No FHCC (n =2580)**				
T1 (< 48.75)	588 (33.7)	401 (47.9)	1.00	1.00
T2 (48.75 - 88.17)	577 (33.1)	289 (34.5)	0.73 (0.61 - 0.89)	0.74 (0.58 - 0.94)
T3 (> 88.17)	578 (33.2)	147 (17.6)	0.37 (0.30 - 0.47)	0.49 (0.38 - 0.65)
p for trend			**< 0.001**	**< 0.001**
**FHCC (n = 185)**				
T1 (< 48.75)	26 (26.3)	42 (48.8)	1.00	1.00
T2 (48.75 - 88.17)	36 (36.4)	17 (19.8)	0.29 (0.14 - 0.62)	0.19 (0.06 - 0.61)
T3 (> 88.17)	37 (37.4)	27 (31.4)	0.45 (0.23 - 0.91)	0.28 (0.09 - 0.84)
p for trend			0.060	0.052

Supplementary table 2. Association between retinol intake and colorectal cancer risk stratified by family history of colorectal cancer of the participants. Model 1: crude model. Model 2: multivariate model, adjusted for age, sex, body mass index (BMI), family history of colorectal cancer, smoking status, alcohol consumption, regular exercise, education, occupation, monthly income, married status, and total energy intake. FHCC: family history of colorectal cancer. T: tertile (μg/day)
